# Deep Learning Method for Mandibular Canal Segmentation in Dental Cone Beam Computed Tomography Volumes

**DOI:** 10.1038/s41598-020-62321-3

**Published:** 2020-04-03

**Authors:** Joel Jaskari, Jaakko Sahlsten, Jorma Järnstedt, Helena Mehtonen, Kalle Karhu, Osku Sundqvist, Ari Hietanen, Vesa Varjonen, Vesa Mattila, Kimmo Kaski

**Affiliations:** 1 Aalto University School of Science, 00076 Aalto, Finland; 2 Medical Imaging Centre, Department of Radiology Tampere University Hospital, Teiskontie 35, 33520 Tampere, Finland; 3 Planmeca Oy, Asentajankatu 6, 00880 Helsinki, Finland; 4 Alan Turing Institute, British Library, 96 Euston Rd, London, NW1 2DB UK

**Keywords:** Machine learning, Cone-beam computed tomography, Medical imaging

## Abstract

Accurate localisation of mandibular canals in lower jaws is important in dental implantology, in which the implant position and dimensions are currently determined manually from 3D CT images by medical experts to avoid damaging the mandibular nerve inside the canal. Here we present a deep learning system for automatic localisation of the mandibular canals by applying a fully convolutional neural network segmentation on clinically diverse dataset of 637 cone beam CT volumes, with mandibular canals being coarsely annotated by radiologists, and using a dataset of 15 volumes with accurate voxel-level mandibular canal annotations for model evaluation. We show that our deep learning model, trained on the coarsely annotated volumes, localises mandibular canals of the voxel-level annotated set, highly accurately with the mean curve distance and average symmetric surface distance being 0.56 mm and 0.45 mm, respectively. These unparalleled accurate results highlight that deep learning integrated into dental implantology workflow could significantly reduce manual labour in mandibular canal annotations.

## Introduction

The human mandible, also known as the lower jaw, is anatomically complex and it is the only movable bone in the facial area facilitating the functions of mastication, speech and facial expressions. It also serves as a scaffold and platform for the lower dentition, muscle insertions, temporomandibular joint, nerves, and vessels. Important mandibular structures are the two mandibular canals that are located bilaterally underneath the teeth in the premolar and molar regions. The mandibular canals have two openings called mandibular foramen in the ramus area, and mental foramen in the parasymphyseal area. Each canal contains the artery and vein as well as the inferior alveolar nerve, which is part of the mandibular branch of the trigeminus nerve that supplies the motor innervations to muscles and sensory innervations to the teeth, chin, and lower lip.

In order to detect and diagnose these anatomical structures, 3D computed tomography (CT) imaging techniques are commonly used. The accuracy of these techniques is affected by the anatomical variability between individuals and variations in radiological visibility. This poses a challenge to locate the mandibular canals with sufficiently high precision to avoid complications in dentomaxillofacial surgical operations. To achieve this, the cone beam computed tomography (CBCT) is widely applied to dentomaxillofacial radiology for 3D diagnostics and operation planning. A clinical alternative is the multi-detector computed tomography (MDCT), though its use is limited by high radiation dosage and low spatial resolution. In contrast, the CBCT allows more accurate imaging of hard tissues in dentomaxillofacial area and its effective radiation dosage is lower than that of the MDCT^1^. In addition, CBCT is cost-efficient and easily available^2^.

Dental implantology is one of the most common surgical operations on jaws. In order to plan the position, place, and size of the implant(s) and how the surgery is to be conducted, the mandibular canals need to be located accurately. A popular approach is to label the canal to cross-sectional slices using 3D imaging software, to produce the segmentation of the canal. The labelling is very labour-intensive and time-consuming, thus there is a need for an automated or semi-automated mandibular canal segmentation system or tool to alleviate the workload on radiologists. Earlier studies had concentrated on 3D CT-scans^3,4^, but more recent works also on 3D CBCT scans^5–9^. There are various differences between these two approaches^10^, and it has been observed that some of the mandibular canal segmentation methods for the CT scans do not straightforwardly translate to those of the CBCT scans^6^.

The most successful class of methods for the automated segmentation of the mandibular canals has been the statistical shape models (SSM), described by Kainmueller *et al*.^5^ and Abdolali *et al*.^9^. However, these two SSM approaches require the training annotations to include segmented mandible bone, which in turn results in the need for additional manual annotation work or development of a robust algorithm for the segmentation of the mandible. The method described by Moris *et al*.^7^ requires the selection of predefined thresholds for images’ grey-scale values that best separate the mandibular canal from other tissue. As the Hounsfield unit scale is not exact in CBCT scans^10^ and it is also highly dependent on the imaging device, we observed that in our heterogeneous dataset there was no robust threshold limits that could be used to implement the method.

Our approach to the segmentation of the mandibular canal is that of deep neural networks. Deep learning^11^, i.e. the training and use of deep neural networks, has recently gained wide popularity in a variety of medical imaging tasks, such as classification^12–14^ and segmentation^15^. A popular neural network architecture for segmentation is the fully convolutional one, which contains only convolutional layers and can produce segmentation maps as an output^16,17^. Our model uses this architecture with 3D convolutional layers performing the neural network convolutions in all three spatial dimensions and it has the ability to learn patterns that are equivariant to translations in the three spatial dimensions. In this study, we show that a fully convolutional deep neural network can accurately segment the mandibular canal from volumetric CBCT scans and outperform the previously presented SSM approaches. We also analyse the performance of the model relative to the anatomical position on the mandibular canal, and how the model performs on samples that have visual ambiguity in the pathway of the canal.

## Results

### Primary test data

Our main results are obtained for a set of 15 CBCT-scans with voxel-level annotations, serving as our primary test data. As the performance measures, we used the Dice similarity coefficient (DSC), mean curve distance (MCD), average symmetric surface distance (ASSD), and robust Hausdorff distance (RHD). The results using the DSC were 0.57 (SD = 0.08) for the left canal and 0.58 (SD = 0.09) for the right canal, while for the MCD measure we obtained 0.61 mm (SD = 0.16) for the left canal and 0.50 mm (SD = 0.19) for the right canal. For the ASSD we obtained 0.45 mm for both the canals (with left SD = 0.12 and right SD = 0.11), and for the RHD the results were 1.40 mm (SD = 0.63) for the left canal and 1.38 mm (SD = 0.47) for the right canal. The full set of results for the primary test data are presented in Table 1.

**Table 1:** The results for the DSC, MCD, ASSD, and RHD measures are presented for the left and right canals, for all the 15 scans in the primary test data.

Scan	VS (mm)	Left				Right			
		DSC	MCD (mm)	ASSD (mm)	RHD (mm)	DSC	MCD (mm)	ASSD (mm)	RHD (mm)
#1	0.2	0.72	0.39	0.27	0.69	0.70	0.43	0.29	1.20
#2	0.2	0.69	0.46	0.28	0.80	0.67	0.37	0.33	0.89
#3	0.2	0.54	0.47	0.50	1.20	0.62	0.35	0.41	0.89
#4	0.2	0.62	0.47	0.41	1.26	0.65	0.37	0.34	0.89
#5	0.2	0.60	0.52	0.38	1.13	0.67	0.29	0.34	1.20
#6	0.2	0.63	0.52	0.37	1.13	0.55	0.61	0.44	1.33
#7	0.2	0.62	0.55	0.37	0.89	0.59	0.47	0.45	1.26
#8	0.2	0.63	0.60	0.40	1.20	0.63	0.35	0.37	0.98
#9	0.2	0.49	0.61	0.67	2.04	0.54	0.64	0.52	1.33
#10	0.2	0.58	0.65	0.50	1.96	0.62	0.29	0.42	1.13
#11	0.2	0.59	0.66	0.44	1.50	0.56	0.67	0.53	2.04
#12	0.4	0.47	0.75	0.50	1.20	0.35	0.85	0.67	2.30
#13	0.2	0.51	0.76	0.71	3.22	0.51	0.38	0.57	1.65
#14	0.4	0.46	0.87	0.48	1.26	0.45	0.84	0.56	2.26
#15	0.4	0.46	0.95	0.50	1.44	0.54	0.62	0.45	1.39
Mean		0.57	0.61	0.45	1.40	0.58	0.50	0.45	1.38
SD		0.08	0.16	0.12	0.63	0.09	0.19	0.11	0.47

The mean and the standard deviation (SD) of the results are shown in the last two rows. Original voxel spacing (VS) shown in the second column.

Compared to the previous state-of-the-art studies of automated mandibular canal segmentation by Kainmueller *et al*.^5^, and Abdolali *et al*.^9^, our model outperformed both of them in the MCD and the ASSD. In comparison to the more accurate results obtained by Abdolali *et al*. (2017), with MCD = 0.92 mm (SD = 0.15) for the left canal and MCD = 0.82 mm (SD = 0.25) for the right canal, our method improved the mean MCD by more than 0.3 mm for both canals. For the ASSD, Abdolali *et al*. (2017), obtained 0.79 mm (SD = 0.22) for the left canal and 0.84 mm (SD = 0.18) for the right canal, while our method improved the ASSD accuracy by 0.34 mm and 0.39 mm for the left and for the right canal, respectively. The full comparison of the results is presented in Table 2.

**Table 2:** Comparison of the primary results to the reported values by Kainmueller *et al*.^5^ and Abdolali *et al*.^9^.

Metric	Ours	Kainmueller *et al*.^5^	Abdolali *et al*.^9^
Left MCD (mm)	0.61 (SD = 0.16)	1.2 (SD = 0.9)	0.92 (SD = 0.15)
Right MCD (mm)	0.50 (SD = 0.19)	1.0 (SD = 0.6)	0.82 (SD = 0.25)
Left ASSD (mm)	0.45 (SD = 0.12)	—	0.79 (SD = 0.22)
Right ASSD (mm)	0.45 (SD = 0.11)	—	0.84 (SD = 0.18)

**Figure 1:**
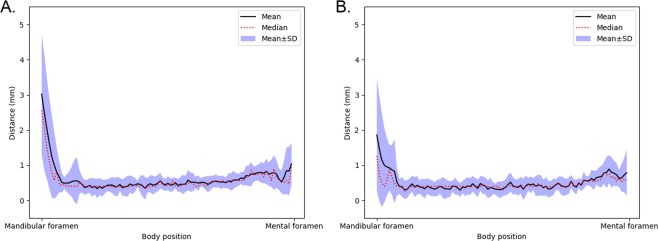
The mean, median, and the area within mean $\pm$ SD of the curve distance calculated on the basis of location from the mandibular foramen to the mental foramen for the primary test data. (**A**) Left canals. (**B**) Right canals.

To analyse the segmentation performance of the model relative to the anatomical position, the curve distance was evaluated at 100 uniformly spaced points for both the canals in the primary evaluation data. The mandibular foramen was selected as the first point and the mental foramen as the last. The relative positional errors for the left and right canals, are illustrated in Fig. 1. The segmentation error can be seen to be relatively large and volatile, near the foramina. However, in the middle part of the canal, the error stabilises to approximately 0.5 mm.

### Secondary test data

As the voxel-level annotations of the primary test data required a large amount of manual labour we have also studied a second set of data, (secondary test data) containing 128 scans with coarsely annotated mandibular canals. The total number of left and right canals was 128 and 124, respectively, and they were annotated using, on average, 10 manually assigned control points, spline interpolation, and a static 3.0 mm canal diameter. In this test there were scans with variable degree of the mandibular canal visibility, which the medical experts had in their coarse annotations graded either as clear or unclear, if the canal could be annotated with high confidence or not, respectively. These grades were used as a basis to divide the secondary test data to “Clear” set, with 86 left and 86 right canals, and “Unclear” set, with 42 left and 38 right canals.

**Figure 2:**
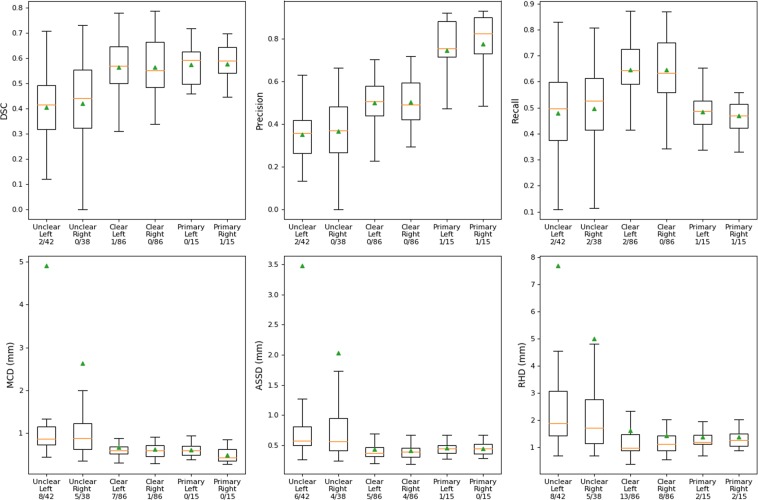
Tukey’s boxplot visualisation of our results for the Clear and Unclear sets as well as for comparison the results for the primary test data. The rectangles contain data within the first and the third quartile. The endpoints of the whiskers are selected as the first quartile −1.5 times the interquartile range (IQR) and third quartile +1.5 IQR. The medians are visualised as orange lines and the means as green triangles. The x-axis label shows the name of the set, the anatomical side of the canal, and the ratio of outliers to the total number of canals. The outliers are defined as the points that are outside the interval defined by the whiskers.

The results for the Clear and Unclear sets are presented in Fig. 2, along with the results of the primary test data, for comparison. The model performance on the larger Clear set using the DSC, MCD, ASSD, and RHD measures turns out to be similar to those obtained for the primary test data. When comparing the Clear set results to those of the Unclear set, the model performance decreases for each of these measures, while at the same time the dispersion increases. The mean values for the DSC, precision, and recall are approximately 0.10-0.15 higher for the Clear set than for Unclear set, for both the left and right canals. For the distance measures, MCD, ASSD, and RHD, the means are significantly larger for the Unclear set, while the medians turn out to be only slightly larger. The fact that the means differ significantly from the medians shows that a few erroneously segmented canals cause the large average errors for the entire Unclear set. This is also illustrated in more detail in the Supplementary Table S1 and Fig. S1, which show that the model performance is poor in few of the more difficult cases, resulting in causing the increased mean values of the MCD, ASSD, and RHD for the Unclear set.

## Discussion

In this study we have shown that a fully convolutional deep neural network model can outperform the previous state-of-the-art results for mandibular canal segmentation. Interestingly, our model produces highly accurate voxel-level predictions for the mandibular canals, even though the data used in model training is as coarsely annotated as the secondary test data. The coarse annotations used many approximations for the canal segmentation, which introduced errors that can be attributed to inaccuracies in labelling, called label noise, against which the deep neural networks are known to have robustness^18^.

As a matter of fact, when the voxel-level annotations are used as the ground truth and the DSC is calculated for the coarse annotations, it turned out to be 0.39 and 0.49 for the left and right canals, respectively. On the other hand, when we calculated the DSC for the model segmentations, the results were 0.57 and 0.58 for the left and right canals. This result shows that the model outperforms the coarse segmentations and the approximation errors do not fully translate to the predictions of the model, thus demonstrating its robustness to the label noise. Full comparison of the coarse segmentations and the voxel-level segmentations are presented in the Supplementary Table S3.

The prediction accuracy of the model turned out to be of the order of 0.5 mm for about 90 percent of the mandibular canal length, which is more than sufficient for dental implantology. Outside this region, near the mandibular and the mental foramina, the prediction accuracy decreases rapidly. This is understandable because clinically the annotation of these two foramen regions is demanding due to the anatomy of the human mandible, and leads to larger approximation errors in the training annotations near the two foramina. However, from the point of view of dental implantology, the decreased performance in the region near the mandibular foramen is less important, since there is no dentition.

As summary, we conclude that an automated deep learning neural network based system when applied to CBCT scans can produce high quality segmentations of mandibular canals. In the future we extend this study to more diverse datasets, including patients’ ethnicity, inaccuracies in labelling, and different CBCT scanners, to evaluate more extensively the robustness of the model under these variabilities. Our results also encourage us to apply deep learning approach in other recognition and segmentation tasks, such as maxillofacial key-point detection and bone density estimation.

## Materials and Methods

### Data

The CBCT dataset consisted of 637 scans from 594 patients. The scans were reconstructed from dentomaxillofacial area CBCT scans ranging from partial scans to whole head scans. The scanning devices were Soredex Scanora 3Dx, (Soredex Oy, Tuusula, Finland) and Planmeca ProMax 3D, ProMax 3D Max, ProMax 3D Mid, (Planmeca Oy, Helsinki, Finland). All the scans had isotropic spatial resolution, with 492 scans with voxel spacing of 0.2 mm, 141 scans with voxel spacing of 0.4 mm, and one scan each with 0.1 mm, 0.15 mm, 0.3 mm, and 0.6 mm voxel spacings. The size of the volumes ranged from $291\times 251\times 251$ voxels to $825\times 825\times 825$ voxels. The CBCT scans had the grey values in the (approximated) Hounsfield unit scale, ranging from -1000 to 3095, except for a few erroneously reconstructed scans with artefacts. The data was scanned in the Cranio and Dentomaxillofacial Radiology Department of The University Hospital of Tampere, Finland. All the acquired CBCT scans were pseudonymized.

The dataset was divided into a training, validation, and test sets, with 457, 52, and 128 CBCT scans, respectively. The model parameters were trained on the training set, the model architecture and hyperparameters selected using the validation set, and the final trained model was evaluated on the test set. In order to avoid overoptimistic results due to over-fitting, that is, memorising patient specific features, each of these sets had a unique set of patients. Moreover, the patients with multiple scans were not included in the validation and test sets. As the number of the 0.1 mm, 0.15 mm, 0.3 mm, and 0.6 mm voxel spacing volumes was very small, they were included in the training set. The scans with the 0.2 mm and the 0.4 mm voxel spacing were randomly divided in similar proportions to the different sets. In addition, each set was constructed to maintain a similar distribution of the two measurement devices. However, all the scans were rescaled to 0.4 mm voxel spacing as a preprocessing step, described later.

In a number of cases the patients’ CBCT scans were taken during preoperative and postoperative radiological examinations, which introduced abnormalities of facial anatomy in the scans of these patients. The scans of the operated patients can also include foreign, mostly metallic material, such as dental implants and fixation materials, causing artefacts of varying degree. Other heterogeneities in the scans include motion artefacts, as well as rotations and translations of the head.

Each scan in the test set was scrutinised for various types of abnormalities. The total number of canals affected by a certain abnormality included 8 cases of osteoporosis, 3 cases of pathological condition, such as benign or malignant tumour, 4 cases of difficult anatomy, 49 cases of difficult bone structure, 9 cases of post bisagittal osteoma operation, 4 cases of metal artefacts, and 11 cases of movement artefacts. Also, 2 of the scans were from cadavers, amounting to 4 canals. The total number of canals affected by heterogeneities was 72, with some of the canals having multiple abnormalities affecting them. Additional results considering model performance for each of these heterogeneities are shown in the Supplementary Table S2.

**Figure 3:**
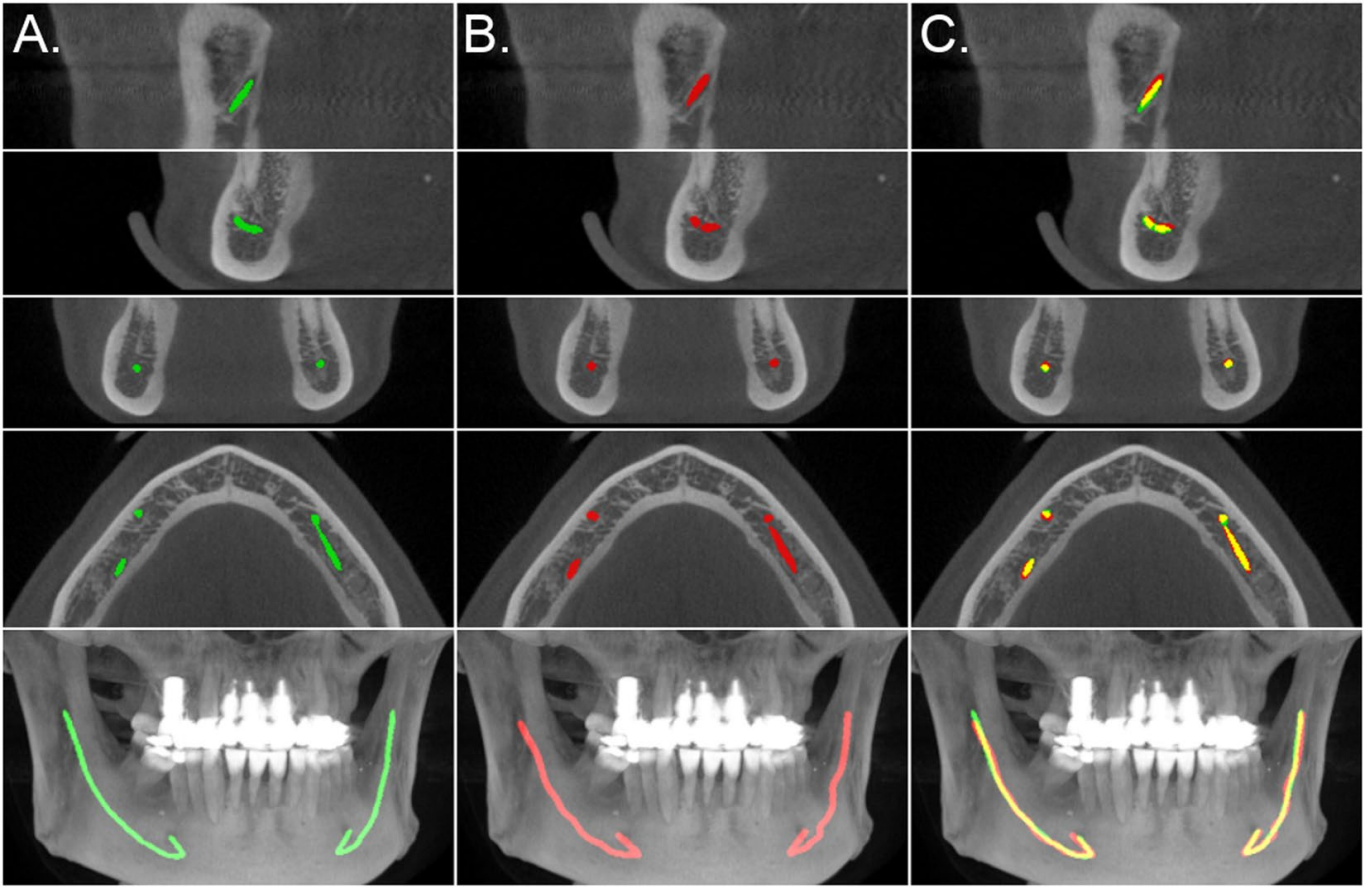
Comparison of the model segmentation and the ground truth, from the secondary test data annotations, for a CBCT scan. (**A**) visualises the segmentation of the model, in green, (**B**) the interpolated control point annotation, in red, and (**C**) the overlap between the model segmentation and the annotation, in yellow. The five rows represent, from top to bottom, a cross section parallel to the sagittal plane centred to the mandibular foramen and the mental foramen, a cross section parallel to the coronal plane and to the axial plane, and a maximum intensity projection (MIP) of the lower region of the head.

The mandibular canals were annotated by two medical professionals. One of them is a dentomaxillofacial radiologist, with 34 years of experience in dentistry, and the other is a resident of dentomaxillofacial radiology, with 10 years of experience in dentistry. These experts annotated the training set and the secondary test data using Romexis® 4.6.2.R software by Planmeca, specifically the built-in tool for mandibular canal annotation. This tool requires the user to specify control points for the canal, and the software interpolates the pathway of the canal based on the control points. The pathway is then expanded to a 3.0 mm diameter tube and finally discretised to provide the ground truth segmentation volumes. This methodology was used in creating the annotations for the training, validation, and test sets. A visualisation of the model segmentation and the annotation is presented in Fig. 3 for a representative scan. Informed consent was obtained from the subject shown in this figure for the publication.

The sparse specification of the control points and the discretisation of the interpolated tube introduced noise to the ground truth annotations. For accurate evaluation, 15 CBCT volumes were selected from the test set and annotated at the voxel level. The selection was based on clear visibility of the mandibular canals, while preserving the distribution of voxel spacings. As such, there was not any canals affected by the abnormalities in the set. Due to the more labour-intensive and time-consuming nature of the voxel-level annotation, these annotations were only made for model evaluation purposes. The software used for the voxel-level annotation was Mimics inPrint 3.0 software (Materialise, Leuven, Belgium). The 15 CBCT scans with voxel-level annotations served as our primary test data. The full test set with coarse annotations was used as the secondary test data.

In order to standardise the data three preprocessing steps were used. First, all the scans were resized to the 0.4 mm voxel spacing using linear interpolation. This procedure reduced significantly the memory footprint of the largest volumes, such as the whole head scans with 0.2 mm voxel spacing. Second, the values outside the valid Hounsfield unit scale were clipped to the valid range of  $[-1000,3095]$. Third, the grey values were then normalised to the interval [0, 1].

### The segmentation model

**Figure 4:**
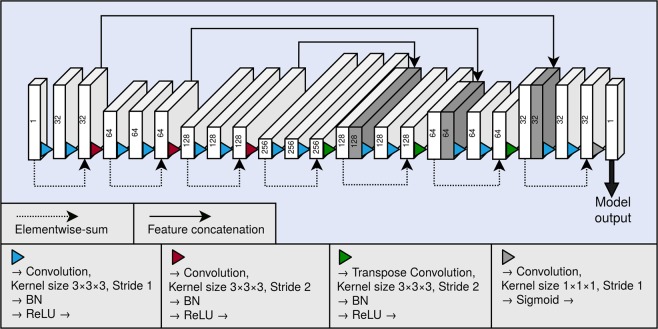
Graphical illustration of our fully convolutional neural network architecture. Each block depicts a hidden layer of the network. The height of each of the blocks visualises the evolution of the spatial size in the network. As a comparison, the depth visualises the evolution of the number of the feature maps in the network. The number of the feature maps of each layer is also shown as a number on each block. The blocks containing concatenation are shown in dark grey. BN and ReLU correspond to the batch normalisation operation and the rectified linear unit non-linearity, respectively.

As the backbone of our segmentation method, we used a 3D fully convolutional neural network. A graphical illustration of the model is presented in Fig. 4. The neural network architecture is similar to the U-net^16^, and can be divided to a contractive pathway and an expanding pathway. In the contractive pathway, the feature maps are down-sampled with stride 2 convolutions, and in the expanding pathway, the feature maps are up-sampled with stride 2 transpose convolutions. With the exception of the last layer of the neural network, all convolutions and transpose convolutions have the kernel size of $3\times 3\times 3$, and are followed by a batch normalisation operation^19^ and a rectified linear unit (ReLU) non-linearity. The last layer of the network has a convolution with the kernel of size $1\times 1\times 1$, and the link function is chosen as the logistic sigmoid. Between the contracting and the expanding pathways are long skip connections, which concatenate the hidden layers along the channel dimension. The network also utilises residual connections^20^ within each of the down-sampling and up-sampling blocks.

The CBCT volumes had spatial dimensions that were too large to fit into a workstation graphical processing unit (GPU), using our model. Thus, a patch sampling method was employed, in which $3{2}^{3}$ sized patches were randomly sampled using a stride of 22. To reduce the class imbalance, the patches without any mandibular canal voxels were not included in the training set. Each of the sampled patches were augmented with random flips in all the spatial dimensions. The model was trained for 400 epochs using a mini-batch size of 24. Parameter updates were calculated using the Adam algorithm^21^, with the learning rate set to 0.0001, ${\beta }_{1}$ as 0.9, and ${\beta }_{2}$ as 0.999.

The network was trained using a differentiable version of the Dice similarity coefficient, which alleviates the class-imbalance problem when calculated for the minority class. The DSC is defined as the intersection of two sets, normalised with the cardinalities of the sets. However, this definition is not differentiable, as it assumes thresholded binary predictions. The Dice loss (DL) used to train our network is based on the soft Dice coefficient objective function, proposed in^17^, and is defined as follows, in the Eq. (1):

1 $${\rm{DL}}=-\,2\frac{{\sum }_{n=1}^{N}\,{t}_{n}{p}_{n}}{{\sum }_{n=1}^{N}\,{t}_{n}+{p}_{n}}.$$

Here ${t}_{n}$ is a binary variable which represents the ground truth label of the voxel indexed by $n$, and is 0 for the negative class and 1 for the positive class. ${p}_{n}$ is the probability that the voxel indexed by $n$ belongs to the positive class, and is determined by the model.

In addition, we developed a post-processing method to refine the model predictions. The raw model output had large quantities of false positives, likely due to sampling the training patches close to the mandibular canal. The output was filtered with a connected-component algorithm that connects neighbouring voxels to larger structures, from which the two largest structures were chosen as the predicted canals. In the model evaluation, the number of canals was known for each volume, and this information was used to select the appropriate number of structures.

### Performance measures

In order to measure the performance of the deep learning model, we calculated the DSC, precision, and recall using the following Eqs. (2)–(4):

2 $${\rm{DSC}}=\frac{2\ {\rm{TP}}}{2\ {\rm{TP}}+{\rm{FP}}+{\rm{FN}}},$$

3 $${\rm{Precision}}=\frac{{\rm{TP}}}{{\rm{TP}}+{\rm{FP}}},$$

4 $${\rm{Recall}}=\frac{{\rm{TP}}}{{\rm{TP}}+{\rm{FN}}}.$$

Here TP denotes the true positives, FP false positives, and FN false negatives.

The DSC, precision, and recall give us insight into the voxel-level segmentation performance of the model. However, these measures do not consider the distance from the predictions to the ground truth canal in the patient’s mandible, which is of key importance for example from the dental surgery point of view. In order to include the location based performance measures, we also evaluate the segmentation performance of our model using the following three distance based measures: average symmetric surface distance (ASSD), mean curve distance (MCD) and robust Hausdorff distance (RHD), calculated using the Eqs. (5), (6), and (7), respectively:

5 $${\rm{ASSD}}=\frac{1}{| S(T)| +| S(P)| }(\sum _{{\boldsymbol{t}}\in S(T)}d({\boldsymbol{t}},S(P))+\sum _{{\boldsymbol{p}}\in S(P)}d({\boldsymbol{p}},S(T))),$$

6 $${\rm{MCD}}=\frac{1}{| C(T)| }\sum _{{\boldsymbol{t}}\in C(T)}d({\boldsymbol{t}},C(P)),$$

7 $${\rm{RHD}}=\max \{\mathop{\max }\limits_{{P}_{95}}d({\boldsymbol{t}},S(P)),\mathop{\max }\limits_{{P}_{95}}d({\boldsymbol{p}},S(T))| {\boldsymbol{t}}\in S(T),{\boldsymbol{p}}\in S(P)\}.$$

Here $d({\boldsymbol{b}},B)={\min }_{{\boldsymbol{b}}\in B}\left\{\parallel {\boldsymbol{a}}-{\boldsymbol{b}}{\parallel }_{2}\right\}$. The ${\boldsymbol{t}}$ denotes the coordinates of a ground truth canal voxel, ${\boldsymbol{p}}$ the coordinates of a predicted canal voxel, $T$ the set of ground truth canal voxel coordinates, $P$ the set of predicted canal voxel coordinates, $S(\,\cdot \,)$ an operation which extracts the surface voxels of a set of voxels, $C(\,\cdot \,)$ an operation which extracts the curve line of a set of voxels, in practice implemented as a skeletonization algorithm, and ${P}_{k}$ denotes the $k$:th percentile.

## Supplementary information

Supplementary Information.

## Data Availability

The datasets used in model training, validation, and testing were provided by TAYS, and as such is not publicly available and restriction apply to their use according to the Finnish law and General Data Protection Regulation (EU).

## Code availability

The codes used for preprocessing and deep learning includes proprietary parts and cannot be released publicly. However, preprocessing and deep learning algorithms can be replicated using the information in the Methods section.
