# Supplementary material for: Deep Learning Method for Mandibular Canal Segmentation in Dental Cone Beam Computed Tomography Volumes
Source: Sci Rep. 2020 Apr 3;10:5842. doi: 10.1038/s41598-020-62321-3 (PMC7125134; doi:10.1038/s41598-020-62321-3)
Supplement: Supplementary file 1 — Supplementary Information. [file 41598_2020_62321_MOESM1_ESM.pdf]

Supplementary for Deep Learning Method for Mandibular Canal Segmentation in Dental CBCT Volumes

Joel Jaskari[1]
joel.jaskari@aalto.fi

Jaakko Sahlsten[1]
jaakko.sahlsten@aalto.fi

Jorma Järnstedt[2]
jorma.jarnstedt@pshp.fi

Helena Mehtonen[2]
helena.mehtonen@pshp.fi

Kalle Karhu[3]
kalle.karhu@planmeca.com

Osku Sundqvist[3]
osku.sundqvist@planmeca.com

Ari Hietanen[3]
ari.hietanen@planmeca.com

Vesa Varjonen[3]
vesa.varjonen@planmeca.com

Vesa Mattila[3]
vesa.mattila@planmeca.com

Kimmo Kaski[*1,4]
kimmo.kaski@aalto.fi

[1]Aalto University School of Science, 00076 Aalto, Finland.
[2]Medical Imaging Centre, Department of Radiology Tampere University Hospital, Teiskontie 35, 33520 Tampere, Finland
[3]Planmeca Oy, Asentajankatu 6, 00880 Helsinki, Finland
[4]Alan Turing Institute, British Library, 96 Euston Rd, London NW1 2DB, UK

[*]corresponding author

1 Supplemenatry Results and Discussion

The results used for the Figure 2 of the main manuscript are presented in Supplementary Table S1. The table also presents results for the primary test data when comparing ground-truth to the unprocessed neural network output without our developed post-processing method. The large quantity of false positives in these unprocessed predictions prevent the use of MCD, ASSD, and RHD, due to no meaningful structures present. The Supplementary Figure S1 is a recreation of the main manuscript Figure 2 with the outliers included. The Supplementary Table S2 presents results for the secondary test data conditional on the heterogeneity affecting the canal.

The Supplementary Table S3 presents comparison between the model and the medical expert, when the medical expert uses the coarse annotation tool, and both are evaluated against the voxel-level annotations. Results show that the model outperforms the medical expert in DSC, ASSD, and RHD. The results are the same for the MCD measure.

		Unclear		Clear		Primary		Primary[a]	
		L	R	L	R	L	R	L	R
Count	N	42	38	86	86	15	15	15	15
Dice	Mean	0.40	0.42	0.56	0.56	0.57	0.58	0.48	0.48
	SD	0.17	0.18	0.11	0.11	0.08	0.09	0.16	0.15
	Median	0.41	0.44	0.57	0.55	0.59	0.59	0.54	0.50
Precision	Mean	0.35	0.37	0.50	0.50	0.75	0.78	0.54	0.53
	SD	0.15	0.16	0.10	0.11	0.16	0.17	0.23	0.23
	Median	0.36	0.37	0.51	0.49	0.76	0.83	0.59	0.59
Recall	Mean	0.48	0.50	0.65	0.65	0.48	0.47	0.48	0.47
	SD	0.20	0.21	0.11	0.12	0.10	0.09	0.10	0.09
	Median	0.50	0.53	0.64	0.63	0.49	0.47	0.49	0.47
MCD	Mean	4.91	2.63	0.67	0.62	0.61	0.50	N/A	N/A
	SD	15.30	7.33	0.37	0.26	0.16	0.19	N/A	N/A
	Median	0.87	0.88	0.61	0.61	0.60	0.43	N/A	N/A
ASSD	Mean	3.48	2.03	0.43	0.41	0.45	0.45	N/A	N/A
	SD	11.11	6.77	0.22	0.17	0.12	0.11	N/A	N/A
	Median	0.57	0.56	0.37	0.39	0.44	0.44	N/A	N/A
RHD	Mean	7.70	5.00	1.62	1.45	1.40	1.38	N/A	N/A
	SD	16.12	10.07	1.87	1.42	0.63	0.47	N/A	N/A
	Median	1.90	1.72	0.98	1.13	1.20	1.26	N/A	N/A

Supplementary Table 1: Mean, standard deviation (SD), and median of the performance measures for the secondary and primary test data. The Clear and Unclear sets refer to the subsets of secondary test data defined in the main manuscript.
[a]Before post-processing

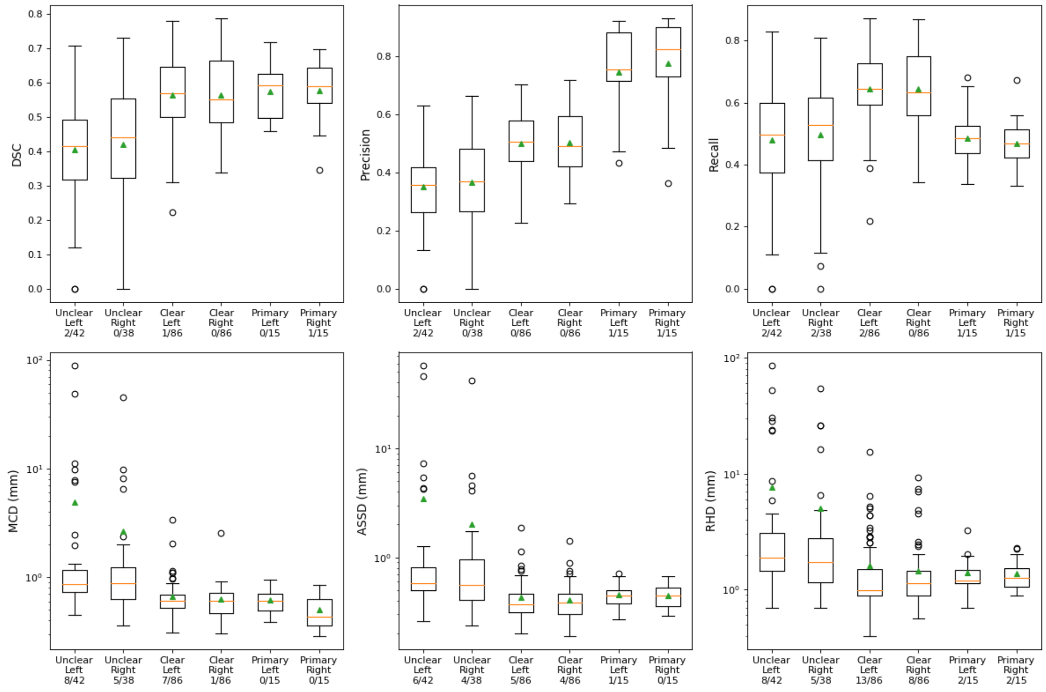

Supplementary Figure 1: Tukey's boxplot visualization of the results for the Clear and Unclear sets. The results for the primary test data are also shown, for comparison. The rectangle contains data within the first and the third quartile. The endpoints of the whiskers are selected as the first quartile − 1.5 times the interquartile range (IQR) and third quartile + 1.5 IQR. The median is visualized as an orange line and the mean as a green triangle. The x-axis label shows the name of the set, the anatomical side of the canal, and the ratio of outliers to the total number of canals. The outliers are defined as the points that are outside the interval defined by the whiskers. Logarithmic y-axis scale is used for the MCD, ASSD, and RHD plots.

Heterogeneity	Negative					Positive				
	Count	DSC	MCD mm	ASSD mm	RHD mm	Count	DSC	MCD mm	ASSD mm	RHD mm
Metal artefact	248	0.52 (0.15)	1.64 (7.04)	1.17 (5.36)	3.02 (8.08)	4	0.34 (0.21)	2.62 (2.74)	1.55 (1.71)	7.08 (6.95)
Movement artefact	241	0.52 (0.14)	1.63 (7.13)	1.16 (5.44)	2.96 (8.08)	11	0.34 (0.15)	2.25 (2.48)	1.37 (1.43)	5.80 (7.53)
Pathological condition	249	0.52 (0.15)	1.63 (7.00)	1.15 (5.34)	2.99 (7.92)	3	0.45 (0.29)	4.10 (6.05)	2.68 (4.02)	10.91 (16.99)
Bisagittal osteoma	243	0.52 (0.15)	1.65 (7.10)	1.17 (5.41)	3.02 (8.07)	9	0.42 (0.16)	1.92 (3.00)	1.12 (1.69)	4.92 (8.02)
Cadaver	248	0.52 (0.15)	1.29 (4.30)	0.94 (3.98)	2.67 (6.02)	4	0.36 (0.25)	24.55 (43.02)	15.73 (27.95)	28.68 (39.63)
Difficult anatomy	248	0.52 (0.14)	1.11 (3.29)	0.77 (2.99)	2.45 (5.05)	4	0.16 (0.19)	35.79 (40.21)	26.26 (27.93)	42.09 (35.72)
Difficult bone structure	203	0.54 (0.13)	0.84 (1.20)	0.53 (0.73)	2.03 (3.62)	49	0.40 (0.18)	5.06 (15.31)	3.83 (11.70)	7.43 (16.15)
Osteoporosis	244	0.52 (0.14)	1.30 (5.80)	0.83 (3.74)	2.69 (6.82)	8	0.23 (0.18)	12.53 (21.05)	11.61 (20.06)	15.03 (23.43)
Any above heterogeneity	180	0.56 (0.11)	0.66 (0.32)	0.42 (0.19)	1.53 (1.62)	72	0.41 (0.17)	4.16 (12.78)	3.05 (9.75)	6.98 (14.20)
Ambiguous (Clear/Unclear)	172	0.56 (0.11)	0.65 (0.32)	0.42 (0.19)	1.53 (1.66)	80	0.41 (0.17)	3.83 (12.16)	3.19 (11.23)	7.27 (15.97)

Supplementary Table 2: Results for the heterogeneity analysis. Each row describes a certain abnormality. The ”Negative” and ”Positive” groups of columns describe results for the canals which are unaffected and affected, respectively, by the abnormality. Results are presented as mean values with standard deviation in brackets.

Metric	Coarse Segmentation	Model Output
Left DSC	0.39 (SD = 0.10)	0.57 (SD = 0.08)
Right DSC	0.43 (SD = 0.05)	0.58 (SD = 0.09)
Left MCD (mm)	0.61 (SD = 0.15)	0.61 (SD = 0.16)
Right MCD (mm)	0.50 (SD = 0.18)	0.50 (SD = 0.19)
Left ASSD (mm)	0.72 (SD = 0.21)	0.45 (SD = 0.12)
Right ASSD (mm)	0.65 (SD = 0.10)	0.45 (SD = 0.11)
Left RHD (mm)	2.99 (SD = 1.74)	1.40 (SD = 0.63)
Right RHD (mm)	2.49 (SD = 1.13)	1.38 (SD = 0.47)

Supplementary Table 3: Comparison of the model output and the medical expert using the annotation tool for the coarse segmentation, for voxel-level annotation on the primary test data. We can see that the model outperforms, or is equal to, the performance of the coarse annotations in each of the performance measures.
